# Stress-Related Mitogen-Activated Protein Kinases Stimulate the Accumulation of Small Molecules and Proteins in *Arabidopsis thaliana* Root Exudates

**DOI:** 10.3389/fpls.2017.01292

**Published:** 2017-07-21

**Authors:** Nadine Strehmel, Wolfgang Hoehenwarter, Susann Mönchgesang, Petra Majovsky, Sylvia Krüger, Dierk Scheel, Justin Lee

**Affiliations:** ^1^Department of Stress and Developmental Biology, Leibniz Institute of Plant Biochemistry Halle, Germany; ^2^Research Group Proteome Analytics, Leibniz Institute of Plant Biochemistry Halle, Germany

**Keywords:** root exudates, roots, metabolite profiling, plant defense, signaling, *Arabidopsis thaliana*, metabolomics

## Abstract

A delicate balance in cellular signaling is required for plants to respond to microorganisms or to changes in their environment. Mitogen-activated protein kinase (MAPK) cascades are one of the signaling modules that mediate transduction of extracellular microbial signals into appropriate cellular responses. Here, we employ a transgenic system that simulates activation of two pathogen/stress-responsive MAPKs to study release of metabolites and proteins into root exudates. The premise is based on our previous proteomics study that suggests upregulation of secretory processes in this transgenic system. An advantage of this experimental set-up is the direct focus on MAPK-regulated processes without the confounding complications of other signaling pathways activated by exposure to microbes or microbial molecules. Using non-targeted metabolomics and proteomics studies, we show that MAPK activation can indeed drive the appearance of dipeptides, defense-related metabolites and proteins in root apoplastic fluid. However, the relative levels of other compounds in the exudates were decreased. This points to a bidirectional control of metabolite and protein release into the apoplast. The putative roles for some of the identified apoplastic metabolites and proteins are discussed with respect to possible antimicrobial/defense or allelopathic properties. Overall, our findings demonstrate that sustained activation of MAPKs alters the composition of apoplastic root metabolites and proteins, presumably to influence the plant-microbe interactions in the rhizosphere. The reported metabolomics and proteomics data are available via Metabolights (Identifier: MTBLS441) and ProteomeXchange (Identifier: PXD006328), respectively.

## Introduction

Plants have developed mechanisms to cope with various abiotic and biotic stresses arising from their changing environment. Phosphorylation via mitogen-activated kinase (MAPK) cascades is a highly conserved eukaryotic signaling mechanism that is important to transduce extracellular stress signals into cellular biochemical responses (Pitzschke et al., 2009). For instance, MAPKs are activated after sensing of molecules derived directly from microbial pathogens or indirectly through damage generated during the infection process. These conserved molecules are termed microbe- and damage-associated molecular patterns (MAMPs and DAMPs), respectively and their recognition subsequently leads to pattern-triggered immunity (PTI) in plants (Li et al., 2016).

**Figure 1:**
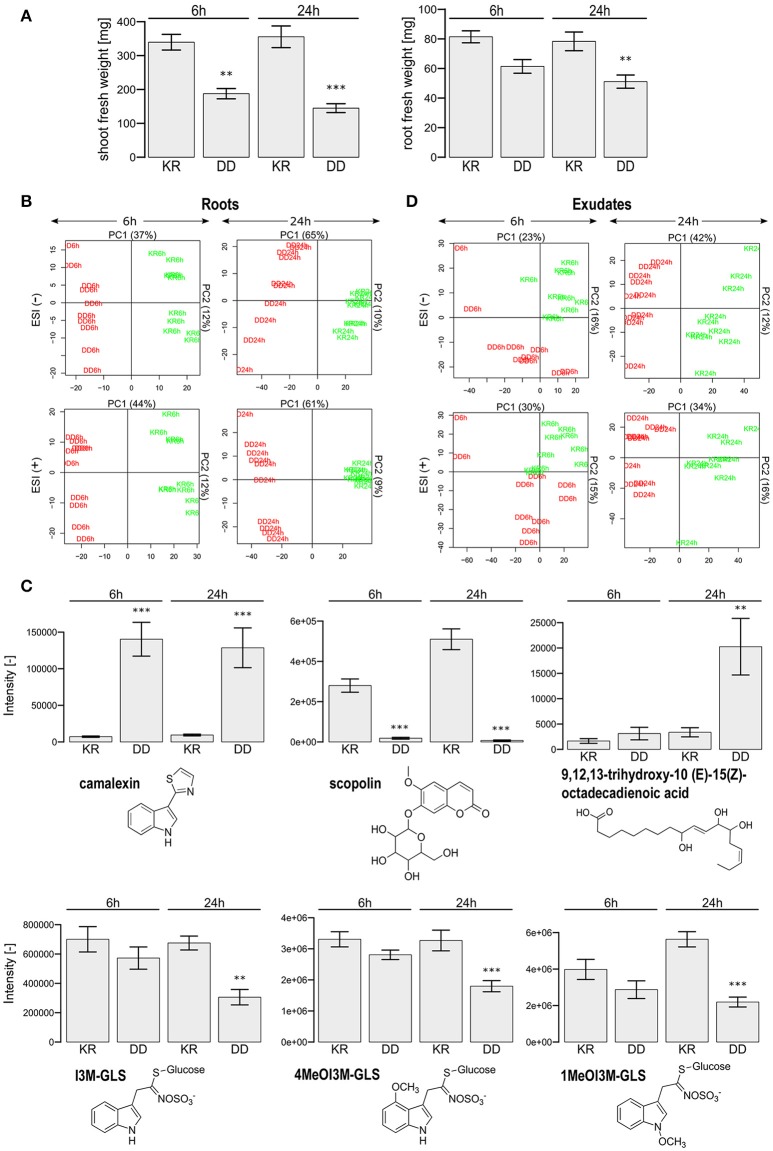
Phenotypic characterization of dexamethasone-treated *Arabidopsis thaliana* Col-0 plants. **(A)** Shown are the shoot and root fresh weight (mean ± SEM) for a pool of four plants. Dexamethasone was applied to the roots of 5-week old plants for 6 or 24 h and transferred to water for 24 h. Student's *t*-test (*p*-value) comparison between the constitutively active MKK5-DD (DD) and the corresponding control (KR) lines: ^**^p < 0.01; ^***^p < 0.001. **(B)** Principal Component Analysis of LC/ESI(+/−)-MS metabolite profiles of roots obtained 6 and 24 h after DEX treatment. The KR and DD samples are color-coded in green and red, respectively. **(C)** Examples of selected compounds with differential levels in the KR and DD roots after 6 and 24 h DEX treatment. Asterisks mark the statistically significant differences (Student's *t*-test comparison to the corresponding KR line; *p*-value: ^**^p < 0.01; ^***^p < 0.001). **(D)** Principal Component Analysis of LC/ESI(+/−)-MS metabolite profiles as described in **(B)** above but for the root exudates. The KR and DD lines are color-coded in green and red, respectively.

MAPK cascades typically involve three hierarchically acting kinases—with the MAPK being phosphorylated and activated by a MAPK kinase (MKK), which in turn needs to be activated by an upstream MKK kinase (M3K). The most downstream MAPK eventually phosphorylates specific substrates to initiate an appropriate cellular response. This may encompass a variety of reactions such as re-localization of the substrate proteins, modulation of their (enzymatic) activity, or alteration of gene expression (Meng and Zhang, 2013). For the latter, this could operate through MAPK substrates that are transcription factors (Tyanova et al., 2016) or regulate post-transcriptional events (Xu and Chua, 2012; Maldonado-Bonilla et al., 2014; Roux et al., 2015). MPK3 and MPK6 are two of four main MAPKs that are activated by MAMPs in *Arabidopsis thaliana* (Bethke et al., 2012; Eschen-Lippold et al., 2012). Numerous studies have shown that expression of constitutively-active MKKs can activate the corresponding downstream MAPKs and initiate defense reactions such as hormone biosynthesis, defense gene expression, or cell death (Ren et al., 2002; Kim and Zhang, 2004; Liu and Zhang, 2004). The tobacco NtMEK2 (Kim and Zhang, 2004) and the parsley PcMKK5 (Lee et al., 2004) are the closest homologs of the Arabidopsis MKK4/5 and replacement of two serine/threonine residues within their activation loop by phospho-mimicking aspartate residues enhances their kinase activity. Through heterologous expression of constitutively-active MKK5 (hereafter abbreviated as MKK5-DD) in Arabidopsis, MPK3 and MPK6, but not the other MAMP-responsive MPK4 or MPK11, are activated (Lassowskat et al., 2014). Since the only known targets of MKKs are MAPKs, expression of constitutively-active MKK5 is equivalent to activation of MPK3 and MPK6 (for details, see Figure 1 and Figure S1 of Lassowskat et al., 2014). Thus, such a transgenic system serves to mimic MAMP-induced responses controlled by MPK3/6, which are activated during infection or stress. By using an inducible promoter to drive the expression of MKK5-DD, one can thus specifically focus on MPK3/6 actions without all the other signaling pathways activated in parallel by pathogens or stress treatments.

Proteome and metabolome analyses with this MKK5-DD transgenic line were employed to study the defense metabolome and to identify downstream MAPK substrates in leaves (Lassowskat et al., 2014). The global proteomics study revealed that primary metabolism, translation, peptidases, proteasome as well as glucosinolate metabolism, tryptophan biosynthesis, and glutathione S-transferases are upregulated as a result of MPK3 and MPK6 activation. On the metabolic level, MPK3/6 activation induced the biosynthesis of camalexin and accumulation of various indole-3-carboxylate derivatives. With regard to indolic glucosinolates (GSL), a depletion of indole 3-ylmethyl GSL (I3M-GSL) in favor of its 4-methoxylated but not its 1-methoxylated variant was noticed, which is a known metabolic shift after flagellin treatment (Clay et al., 2009). Met-derived GSLs did not show altered abundance but accumulated as their respective precursor amino acids and degradation products. Likewise, accumulation of agmatine conjugates and aromatic amino acids was induced upon MPK3/6 activation. However, coumarins and flavonoids seem not to be related to this defense response pathway as is known from abiotic stress (Fourcroy et al., 2014; Schulz et al., 2015; Ziegler et al., 2016). Similarly, the concentration of monolignols and hydroxycinnamates was not affected by MPK3/6 activation. As several of the compounds have antimicrobial activities, a main interpretation of this work is that the sustained MPK3/6 activation is sufficient to drive production of defense metabolites. Since the metabolite accumulation occurs in the absence of pathogen infection, it must be controlled by the actions of the corresponding MAPK substrates (Lassowskat et al., 2014).

Interestingly, multiple components for secretory processes are over-represented among the putative phosphoproteins detected after MPK3/6 activation (Lee et al., 2015). These include proteins from the ER/GOLGI apparatus, vesicle transport, and exocyst complex. Together with the upregulation of defense metabolism in this transgenic system, the question arises whether this is accompanied by an increase in secretory activities, which may be reflected in defense metabolites secretion—possibly as cargo within vesicles through exocytosis. To analyze this, we employed the well-established root exudate system (Strehmel et al., 2014) to monitor secreted metabolites upon MAPK activation. The advantage over standard leaf apoplastic fluid analysis is that no stressful treatments like vacuum infiltration and centrifugations steps are involved, which could lead to MAPK activation and thus confound the findings. In parallel, since most vesicle cargoes are proteins or peptides, we also performed a proteomics analysis in the root exudates. Our results support the notion that activation of the MAPKs, MPK3, and MPK6, can drive the appearance of dipeptides, defense-related metabolites and proteins in the apoplastic fluid of roots. As there is normally a higher exposure to microbes in the rhizosphere compared to the phyllosphere, MAPK-mediated production and secretion of compounds may be a crucial counteracting defense mechanism in roots.

## Materials and methods

### Plant material and growth

The transgenic lines (Lassowskat et al., 2014) with dexamethasone(DEX)-inducible expression of constitutively-active MKK5-DD (line DD55-4) or its corresponding kinase-inactive MKK5-KR variant (line KR51-6) were used. These are abbreviated subsequently as DD or KR, respectively, and were cultivated in a hydroponic system under short-day conditions (8 h day light, 200 μE, 23°C) as described (Strehmel et al., 2014). Briefly, seeds were sown onto agar filled PCR tubes (with the bottom cut off) and placed into a tip box filled with 0.5x MS and 1% sucrose (w/v). After 3 weeks, plantlets were transferred to brown Duran flasks, which had been filled to capacity with modified MS medium (Von Wiren et al., 1995). Twelve flasks were arrayed into an “araponics” growth tray (http://www.araponics.com/) and both genotypes cultivated separately (see Figure S1). The medium was exchanged once (after 7 days) and plants grown for two further weeks prior to DEX treatment. To exclude position effects within the growth chamber, the araponics trays are shifted every second day to a new position to ensure equal illumination during the growth period.

### Dexamethasone treatment

For DEX treatment, plants were transferred to a DEX solution (20 μM in H_2_O, w/v), which had been filled into another set of brown Duran flasks in the araponics growth trays. In a preliminary experiment, we investigated an earlier time point (2 h Dex treatment-24 h exudation), but did not observe a significant effect in the root biomass (*p* = 0.43), only in the shoot biomass (*p* = 0.02) in the course of two independent experiments with three biological replicates each. Thus, based on previous experience with this inducible system, 6 or 24 h of DEX treatment was chosen. Subsequently, plants were transferred to further brown Duran flasks, which had been filled to capacity with water, and root exudates (medium from four flasks) collected for 24 h, under the same conditions as in the growth period. The experiments were conducted four times with 2 (experiment 1 and 2) or 3 (experiment 3 and 4) replicates each. Each replicate is the pooled sample of four individual plants randomly selected from 12 plants. Thus, 40 root as well as 40 root exudate samples [experiment × genotype × dexamethasone treatment period × replicate = 4 × (KR, DD) × (6, 24 h) × (2–3)] were subjected to LC/MS-based metabolite profiling and measured at positive [ESI(+)] and negative [ESI(−)] ionization. In total, 80 root and 80 root exudate profiles were subjected to non-targeted metabolite data analysis.

### Extraction of metabolites from root exudates

For root exudate analysis, the nutrient solutions of four flasks were combined, spiked with 50 μL internal standard (50 μg/mL 2,4-dichlorophenoxyacetic acid) and evaporated until dryness (35 mbar, 40°C) using a rotary evaporator. Then, the residue was reconstituted twice in 3 mL methanol, sonicated for 3 min in an ultrasonic bath, and the unified solution dried down in a glass vial under reduced pressure using a vacuum centrifuge (40°C). Next, the residue was reconstituted in 500 μL methanol, the solution sonicated (3 min) and transferred to a 1.5-mL polypropylene tube. Finally, the supernatant was evaporated in a vacuum centrifuge again and reconstituted in 100 μL methanol/water (30/70; v/v) prior to LC/MS analysis.

### Extraction of metabolites from roots

Root material was homogenized under liquid nitrogen and 200 μL pre-cooled methanol/water (80/20; v/v) were added to 40 mg powdered material. After reaching room temperature, samples were sonicated (10 min, 20°C), centrifuged at 11,200 × g (10 min, 16°C), the supernatant transferred into a new 1.5 mL polypropylene tube, and the residue extracted a second time with 200 μL methanol/water (80/20; v/v). Finally, both extracts were combined and evaporated under reduced pressure at 40°C using a vacuum centrifuge. For LC/MS analysis, the residue was reconstituted in 100 μL methanol/water (30/70; v/v) spiked with internal standard [indole-3-acetyl-L-valine (1 μM), o-anisic acid (1 μM), and kinetin (2 μM)].

### LC/MS-based metabolite profiling

Samples were analyzed by UPLC/ESI(+/−)-QTOF-MS according to Strehmel et al. (2014). For this purpose, an Acquity system from Waters (www.waters.com) and a micrOTOF-Q I equipped with an Apollo II electrospray ion source from Bruker Daltonics (www.bruker.com) were used. All technical details can be found in the supplementary material (Methods section). For non-targeted data evaluation, all raw data files were converted to mzML with the help of CompassXPort version 1.3.10 (Bruker Daltonics) and arranged into four sample classes (KR/6, KR/24, DD/6, DD/24 h). Feature detection was performed using the centWave algorithm [sntresh = 3, prefilter = (3, 100), ppm = 25, peak width = (5, 12)] and alignment accomplished with the help of the XCMS functions *group.density* (minfrac = 1, bw = 2 and mzwid = 0.05), *retcor* (span = 1, missing = 0, extra = 0), and *group.density* (minfrac = 1, bw = 1 and mzwid = 0.03). Missing feature intensities were filled using the XCMS function *fillPeaks*. The intensity matrix was log2-transformed, batch corrected with surrogate variable analysis (Leek and Storey, 2007) and subjected to two Two-tailed Student's *t*-Tests (6 and 24 h). In general, all statistical analysis was either performed with the R statistical language version 3.2.0 (R core team), the Bioconductor environment (Gentleman et al., 2004), the package pcaMethods (Stacklies et al., 2007) or Microsoft Excel. Compounds were annotated based on accurate mass (Δm/z = 0.02) and retention time (Δt = 0.2 min) on the basis of detailed MS characterization of metabolites as previously published (Lassowskat et al., 2014; Strehmel et al., 2014) and manually quantified using QuantAnalysis 2.0 (Bruker Daltonics). For acquisition of MS/MS spectra appropriate ions were selected in the Q1 and fragmented inside the collision cell using argon as collision gas. Product ions were detected as described above. Details of the MS/MS characterization including the MSI levels (Sumner et al., 2007) are summarized in Table S3.

### Proteome analysis of root exudates

A total of 24 root exudates nutrient solutions [experiment × genotype × dexamethasone treatment period × replicate = 2 × (KR, DD) × (6, 24 h) × 3] were analyzed using an LC-MS discovery proteomics approach. Solutions (samples) were filtered through a 150 mL Rapid-Flow Filter unit equipped with a 0.45 μm SFTCA membrane (Thermo Fisher Scientific Nalgene) and subsequently lyophilized until a volume of ~2 mL remained and then transferred to 5 mL reaction tubes. Samples were heated and kept at 70°C for 10 min to aid in denaturation of proteins. 2.5 μl of 200 mM dithiothreitol (DTT) were added and the samples were gently agitated for 1 h at 22°C to reduce cysteine bonds. 10 μL of 200 mM iodoacetamide (IAA) were added to alkylate free sulhydryl groups as above followed by addition of 10 μL of DTT as above to quench the reaction. The pH was adjusted to 7 by addition of 100 μL of 100 mM NH_4_HCO_3_ (pH 8.5). 2 μg of trypsin were added to digest proteins overnight at 37°C. Samples were filtered using a 10 kDa Amicon device (Merck Chemicals GmbH) according to the manufacturer's instructions sequentially loading 400 μL of sample until the entire volume was filtered. The filtrate was dried down in a vacuum concentrator. Dried peptides were dissolved in 0.1% formic acid in ddH_2_O and desalted as previously described (Majovsky et al., 2014). Desalted peptides were dried down again and dissolved in 10 μL 5% acetonitrile (ACN), 0.1% trifluoroacetic acid (TFA) in ddH_2_O.

Peptides were injected into an EASY-nLC1000 UPLC system and separated using reverse phase chemistry using an Acclaim PepMap 100 pre-column (length 2 cm, inner diameter 75 μm, particle diameter 3 μm) in-line with an EASY-Spray ES803 column (length 50 cm, inner diameter 75 μm, particle diameter 2 μm) (both from Thermo Fisher Scientific). Peptides were eluted with a 120 min gradient increasing from 5 to 40% ACN in 0.1% FA, and a flow rate of 250 nL/min and electrosprayed into a QExactive Plus mass spectrometer (Thermo Fisher Scientific) with an EASY-Spray ion source (Thermo Fisher Scientific). The spray voltage was 1.9 kV, the capillary temperature 275°C and the Z-Lens voltage 240 V. A full MS survey scan was carried out with chromatographic peak width set to 15 s, resolution 70,000, automatic gain control (AGC) 3E+06 and a max injection time (IT) of 200 ms. MS/MS peptide sequencing was performed using a Top10 DDA scan strategy with HCD fragmentation. MS/MS scans were acquired with resolution 17,500, AGC 5E+04, IT 150 ms, isolation width 1.6 m/z, normalized collision energy 28, under fill ratio 3%, dynamic exclusion duration 40 s, and an intensity threshold of 1E+04.

Peptides and proteins were identified using the Mascot software v2.5.0 (Matrix Science) linked to Proteome Discoverer v1.4 (Thermo Fisher Scientific). The TAIR10 database (35,394 sequences, 14,486,974 residues; www.arabidopsis.org) with common contaminants (trypsin, human keratin) added was searched with two search nodes with the enzyme set to trypsin and no enzyme tolerating two missed cleavages and unspecific cleavage, respectively. A precursor ion mass error of 5 ppm and a fragment ion mass error of 0.02 Da were tolerated. Carbamidomethylation of cysteine was set as a fixed modification; oxidation of methionine as a variable modification. Search results were imported into the Scaffold software (Proteome Software Inc.) wherein 1% peptide and protein FDR thresholds were applied. The proteins were quantified using the total number of PSMs per protein quantitative index (PQI). The matrix containing the PQI of every protein surpassing the FDR thresholds in every sample was imported into the Perseus software (Tyanova et al., 2016). Samples were categorized in four groups as above for metabolites (KR/6, KR/24, DD/6, DD/24 h). Proteins that were quantified in <50% of measurements of at least one of the four categories were discarded. ANOVA applying a permutation based FDR threshold (250 randomizations) of 5% was performed and the abundance of proteins with *q*-values below said α = 0.05 were considered to change significantly between the groups. PQI values of these proteins were *z*-score transformed and clustered using a combined k-means/hierarchical cluster analysis employing Pearson correlation to produce the sample and Spearman correlation to produce the protein dendrogram.

## Results

### Phenotype after dexamethasone treatment

Dexamethasone(DEX)-inducible expression of the constitutively active MKK5-DD (abbreviated as DD), but not the kinase-inactive MKK5-KR variant (abbreviated as KR), led to a reduced shoot biomass of *A. thaliana* (Lassowskat et al., 2014). Expression of the transgene was induced 4–6 h after spraying DEX onto leaves of soil-grown plants. As we are primarily interested in the roots for the current analysis, we chose to directly apply DEX to the liquid medium of the hydroponics system used here in this report. After some preliminary tests (see Section Dexamethasone Treatment), 6 or 24 h of DEX induction (followed by 24 h of exudation) was chosen for the investigation of the belowground defense response. Roots responded with reduced biomass (Figure 1A). DEX is known to be mobile in plant tissues and can lead to systemic induction distal to the DEX application point (Aoyama and Chua, 1997; Geng and Mackey, 2011). Correspondingly, as expected for efficient transport of solutes through the transpiration stream, the aboveground fresh weight of leaves/shoots was strongly reduced by 6 h of treatment to roots. Surprisingly, although DEX was applied to the roots, reduction of root biomass was less pronounced (or delayed) compared to the leaves/shoots. The root biomass decreased by 1.3-fold after 6 h and 1.7-fold after 24 h DEX treatment, the shoot biomass decreased by 1.9- and 2.6-fold after 6 and 24 h, respectively (Figure 1A). Some curling and drying out of the leaves were observed in the DD plants already with 6 h DEX treatment (Figure S1), which may be the onset of cell death, as seen previously in leaves ~48 h after direct DEX application (Lassowskat et al., 2014). Two possibilities may explain the stronger effect in above ground tissues in the current system. One is that the continued transport through the transpiration stream may hyperaccumulate DEX in the leaves. The second is that the reduced biomass is related to cell death, and since defense-related cell death is often light-dependent (Danon et al., 2006), the effect is stronger in the light-exposed leaf tissues compared to roots. Regardless of these possibilities, the current focus is the root exudates; we therefore proceeded to measure the total root and exudate metabolome.

### Changes in root metabolism

A Principal Component Analysis (PCA) of the root metabolome clearly separated the control (KR) plants from the DD plants after 24 h DEX treatment, with 70% variance for the ESI(+) profile and 75% variance for the ESI(−) profile if one only considers the first two components (Figure 1B). The 6 h time point resulted in similar findings [ESI(+): 56% and ESI(−): 49%). Student's *t*-tests (*p* < 0.01) revealed 347 upregulated (DD/KR > 2-fold; ESI(+): 209 and ESI(−): 138] and 675 downregulated [DD/KR < 0.5-fold; ESI(+): 414 and ESI(−): 261] features after 6 h DEX treatment. For the 24 h DEX treatment, 473 upregulated [ESI(+): 285; ESI(−): 188) as well as 1,577 downregulated (ESI(+): 830; ESI(−): 747] features were found. These could be assigned to 33 differentially regulated compounds (see Table S1).

As reported previously for leaves (Lassowskat et al., 2014), many defense-related metabolites accumulated to a high level in the DD roots, while no changes were observed for flavonol glycosides. Comparable changes were observed in roots for camalexin, coumaroyl agmatine, a set of aliphatic glucosinolate degradation products, and I3CH_2_NH_2_ (Figure 1C, Table S1). In contrast to the previous experiment involving DEX application to leaves (Lassowskat et al., 2014), indolic glucosinolates (I3M-GLS, 4MeOI3M-GLS, and 1MeOI3M-GLS), aliphatic glucosinolates (e.g., 7MeSO-Heptyl GLS, 8MeS-Octyl GLS, 8MeSO-Octyl GLS) and derivatives of indole-3-carboxylic acid (1MeO-I3CH_2_NH_2_, I3CO_2_H), were decreased in direct DEX treatment of the roots of DD plants. The same also holds true for defense-related phytohormones, such as salicylic acid glucosides (SAG), 2,5 dihydroxybenzoic acid (DHBA) pentose, and 2,3-DHBA 3-xylose. Moreover, typical root metabolites, such as cinnamoyl alcohols (coniferin, syringin), coumarins (scopoletin, scopolin, esculetin, esculin), and oligolignols [G(8-5)G, lariciresinol, lariciresinol hexose] were decreased in concentration after DEX treatment, whereas a couple of fatty acids increased in concentration in DD roots (Figure 1C, Table S1).

Interestingly, accumulation for the aromatic amino acids Tyr and Trp was observed only 6 h, but not 24 h, after DEX treatment of the DD line (Table S1). This may reflect increased activity of downstream biosynthetic pathways during the extended DEX treatment period and enhanced flux into subsequent metabolic derivatives. Altogether, the DEX-induced MAPK activation caused an alteration of the root metabolome and the next step was to assess if there is also increased metabolite exudation.

### Non-targeted metabolite profiling of roots exudates

**Figure 2:**
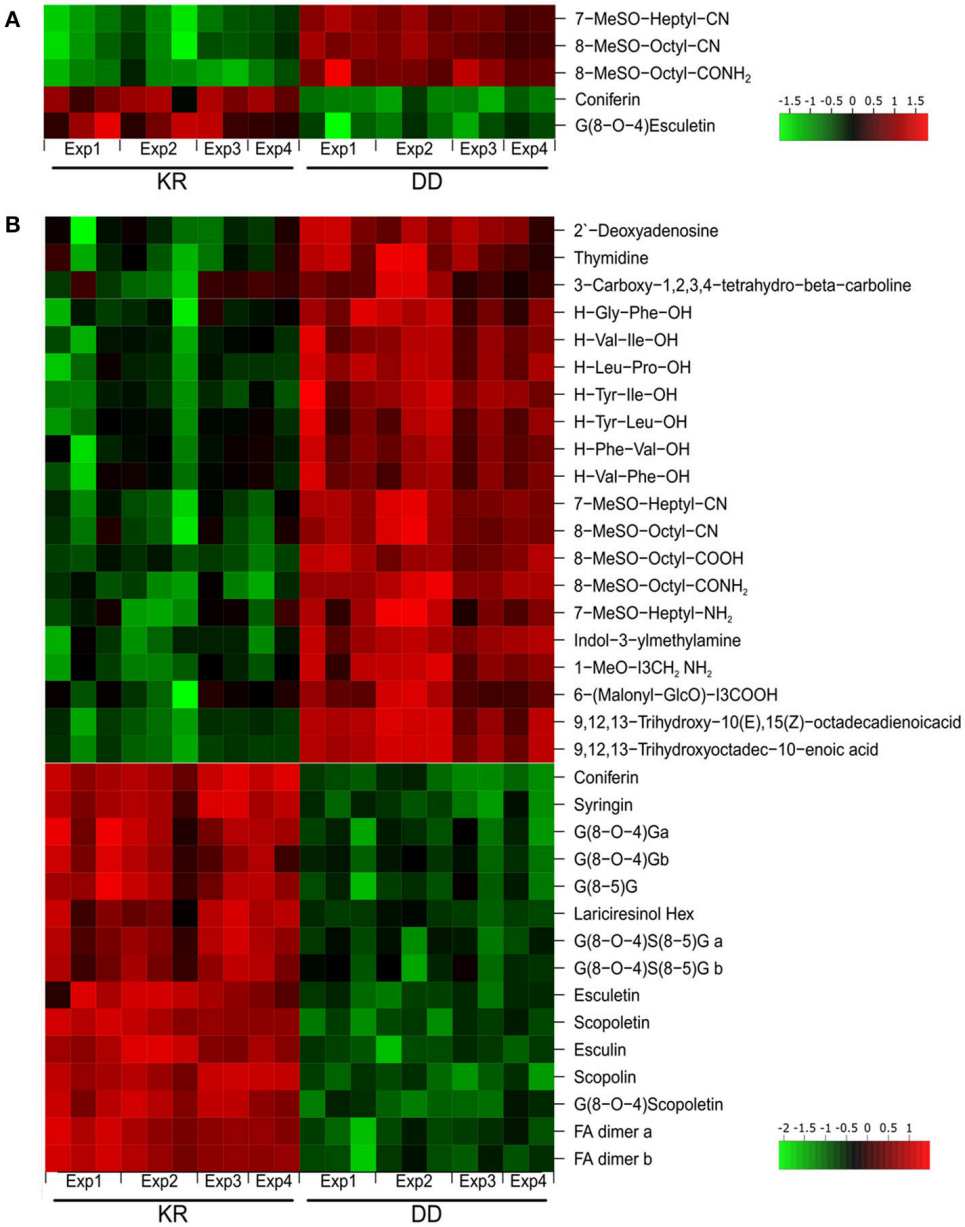
Heat map representing abundance of the root exudate metabolites with differential accumulation (*p* ≤ 0.01). Metabolites in root exudates of KR and DD plants after 6 h **(A)** or 24 h **(B)** of dexamethasone treatment and exudation for 24 h in water. Results from all four independent experiments (Exp) are shown. Intensity values were log_2_-transformed, batch-corrected and Z-scored row-wise. Green, minimal intensity; red, maximal intensity.

A non-targeted LC/MS-based metabolite profiling study of the root exudates revealed a clear separation between the KR and DD genotypes especially for the 24 h DEX treatment. The PCA showed a 50% variance for the ESI(+) profile and 54% for the ESI(−) profile for the 24 h treatment and 45% [ESI(+)] as well as 39% [ESI(−)] for the 6 h DEX treatment if one considers the first two components (Figure 1D). In total, 582 features were increased and 170 features decreased in concentration in the DD plants after 24 h DEX treatment (*p* < 0.01). In contrast, only 142 “upregulated features” as well as 67 “downregulated features” were observed after 6 h treatment. Similar effects were observed for the ESI(+) profile (6 h: 199 features up- and 64 features down-regulated; 24 h: 532 features up- and 169 features down-regulated). Sixty-one compounds could be annotated on the basis of previous MS data (Lassowskat et al., 2014; Strehmel et al., 2014) and current MS/MS validation (Table S3), of which 31 components were differentially regulated after 24 h DEX treatment (Figure 2, Table S2). Generally, there is ~50% of up- or down-regulated substances in the exudates of the DEX-treated DD line. Among the down-regulated substances are phenylpropanoids such as coniferin, and G(8-*O*-4)esculetin, which were reduced after both 6 h (Figure 2A) and 24 h (Figure 2B) DEX treatment. Conversely, the long-chain aliphatic GLS degradation products 7-MeSO-heptyl-CN, 8-MeSO-octyl-CN, 8-MeSO-octyl-CONH_2_, were up-regulated after 6 and 24 h DEX treatment in the exudates of the DD line (Figure 2, Table S2). Taken together with the decreased levels in the total roots of the DEX-treated DD plants (*c.f*. Table S1), the increased detection of long chain aliphatic GLS degradation products in the exudates suggests that these are presumably released into the rhizosphere rapidly.

Furthermore, the levels of several dipeptides (H-Gly-Phe-OH, H-Val-Ile-OH, H-Leu-Pro-OH, H-Tyr-Ile-OH, H-Tyr-Leu-OH, H-Val-Phe-OH, H-Phe-Val-OH), indolic glucosinolate degradation products (I3CH_2_NH_2_, 1-MeO-I3CH_2_NH_2_), as well as fatty acids (9,12,13-trihydroxy-10,15-octadecadienoic acid, 9,12,13-trihydroxyoctadec-10-enoic acid) were increased in root exudates after 24 h DEX treatment (Figure 2B). These components were not affected after 6 h of DEX treatment. By contrast, for compounds of phenylpropanoid metabolism [ferulic acid (FA) dimer, syringin], including coumarins (scopoletin, scopolin, esculetin, esculin), and oligolignols [G(8-O-4)G, G(8-5)G, G(8-O-4)S(8-5)G, G(8-O-4)esculetin, lariciresinol hexose], the levels were reduced after 24 h DEX treatment in the DD line (Figure 2B).

Altogether, the transgenic system used in this study to specifically induce MPK3/6 activation leads to increase of several apoplastic metabolites (including dipeptides) but also down-regulates the release of other compounds into the apoplast.

### Root apoplast proteome

**Figure 3:**
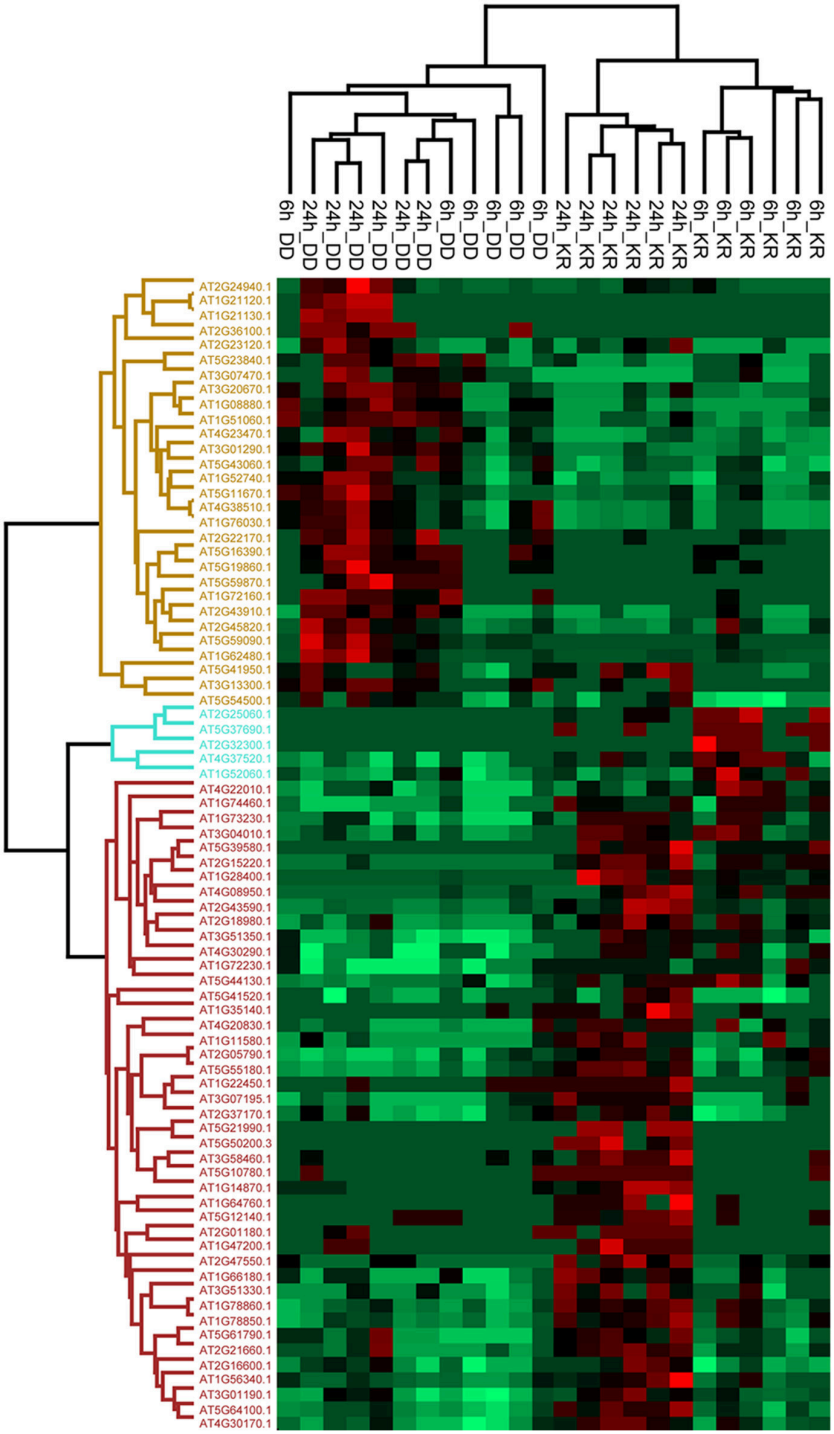
Hierarchical clustering (heat map) showing proteins differentially detected in the root exudates of the DD and KR plant lines after 6 or 24 h of DEX treatment (*q* < 0.05). Each row represents the *Z*-score transformed total number of peptide spectral matches (PSMs) of each protein. Green, minimal abundance; red, maximal abundance. Information about proteins up-regulated in the apoplast is shown in Table 1 (Complete list of all proteins and details on statistics as well as raw values are found in Table S4).

Besides the numerous dipeptides, we asked if larger peptides/proteins could also be found in the root apoplast. Filtered exudate solutions (from independent experiments similar to the set-up described above for metabolome analysis) were lyophilized, tryptic-digested and subjected to LC-MS-based proteome analysis. A total of 78 proteins were identified, with 29 showing up- and 49 down-regulated abundance in exudates of the DEX-treated DD line (Figure 3, Table S4). Most of the up-regulated candidates were detected only after 24 h DEX treatment and not or only barely after 6 h DEX, suggesting a slow release of proteins into the apoplast. Similarly, the majority of the down-regulated proteins (i.e., increased in levels in the KR line) were also detected in the 24 h sample (Figure 3). The detection of both up- and down-regulated proteins after DEX treatment suggests that MPK3/6 activation regulates the appearance of apoplastic proteins both positively and negatively. It also argues against that cell rupture arising from dying tissues might cause random release of proteins/peptides into the root apoplast but rather that secretion is tightly regulated.

As our working hypothesis is increased exocytosis after MPK3/6 activation, we will focus here on the proteins with increased abundance in the root apoplast after DEX-treatment of the DD line. Twenty-six out of these 29 proteins are known extracellular or plasma membrane proteins (Table 1, Table S4). Examples include known secreted proteases (RD21 or subtilase) (Shindo et al., 2012), lipid raft components (remorin) or Hypersensitivity Induced Reaction 2 (HIR2). Notably, HIR proteins (Qi et al., 2011) and remorins (Jarsch and Ott, 2011) have been implicated as membrane microdomains acting as docking platforms for immune signaling (receptor) complexes. This could indicate an increase of signaling complexes at the cell periphery after MPK3/MPK6 activation. While some of the apoplastic proteins are normally classified as intracellular proteins, such as Varicose (AT3G13300) or NADP-malic enzyme 2 (AT5G11670), they have recently been found in extracellular/cell wall or plasma membrane proteomes (De Michele et al., 2016; Nguyen-Kim et al., 2016). In particular, these also include six members of the Histone 2A family (Table 1), which are typically part of the core nucleosomes (Mitra et al., 2009; Heard et al., 2015; Nguyen-Kim et al., 2016). However, other histones such as Histone 4 have previously also been reported in pea root cap secretome (Wen et al., 2007), suggesting possible extracellular functions of histones. Also among the apoplastic proteins is the tetratricopeptide repeat protein, HLB1 (Hypersensitive to Latrunculin B 1, AT5G41950), that is known to control exocytosis (Sparks et al., 2016), thus supporting that secretory processes are stimulated in this transgenic system with activated MPK3/6.

**Table 1:** List of proteins with increased abundance in apoplast after DEX treatment of the DD transgenic line.

**AGI code**	**Annotation**	**Localization**	**Evidence (predicted or MS publication^a^)**
AT5G43060	Secreted papain-like Cys protease RD21	Extracellular/vacuolar	Nguyen-Kim et al., 2016
AT5G59090	Subtilase 4.12	Extracellular/nuclear	Nguyen-Kim et al., 2016
AT2G22170	Lipase/lipooxygenase, PLAT/LH2 family protein	Extracellular/PM	De Michele et al., 2016
AT2G36100	Uncharacterised protein (UPF0497), trans-membrane plant subgroup	Extracellular	Nguyen-Kim et al., 2016
AT3G07470	Protein of unknown function, DUF538	Extracellular (secreted)	Charmont et al., 2005; Nguyen-Kim et al., 2016
AT5G19860	Protein of unknown function, DUF538	Extracellular	Nguyen-Kim et al., 2016
AT5G23840	MD-2-related lipid recognition domain-containing protein	PM/extracellular	predicted by SUBA
AT1G72160	Sec 14p-like phosphatidylinositol transfer family protein	PM	De Michele et al., 2016
AT2G45820	Remorin family protein	PM	De Michele et al., 2016
AT3G01290	Hypersensitive induced reaction (HIR2) proteins	PM	De Michele et al., 2016
AT2G23120	Group 6 LEA (Late embryogenesis abundant) protein	PM	De Michele et al., 2016
AT2G24940	Membrane-associated progesterone binding protein 2, MAPR2	PM	De Michele et al., 2016
AT4G23470	PLAC8 family protein	PM/vacuole membrane	Mitra et al., 2009
AT5G54500	FQR1 (flavodoxin-like quinone reductase 1)	PM/extracellular	deDe Michele et al., 2016
AT4G38510	ATPase, V1 complex, subunit B protein	PM	Heard et al., 2015; De Michele et al., 2016
AT1G76030	ATPase, V1 complex, subunit B protein	PM	Heard et al., 2015; De Michele et al., 2016
AT2G43910	HOL1 (HARMLESS TO OZONE LAYER 1)	PM	De Michele et al., 2016
AT5G41950	Tetratricopeptide repeat (TPR)-like superfamily protein	PM/Golgi	Zhang and Peck, 2011
AT5G11670	NADP-malic enzyme 2	Plastids/PM	Helm et al., 2014; De Michele et al., 2016
AT1G62480	Vacuolar calcium-binding protein-related	Plastids/PM	De Michele et al., 2016
AT3G13300	Varicose, Transducin/WD40 repeat-like superfamily protein	Cytoplasm, P-body/PM	Zhang and Peck, 2011
AT1G08880	HTA5, γ-H2AX, Histone superfamily protein	Nucleus/PM/extracellular	Mitra et al., 2009; Nguyen-Kim et al., 2016
AT5G59870	HTA6, Histone H2A 6	Nucleus/extracellular	Nguyen-Kim et al., 2016
AT1G51060	HTA10, Histone H2A 10	Nucleus/extracellular	Nguyen-Kim et al., 2016
AT1G52740	HTA9, Histone H2A protein 9	Nucleus/multivesicular body	Heard et al., 2015
AT3G20670	HTA13, Histone H2A 13	Nucleus/extracellular	Nguyen-Kim et al., 2016
AT1G21120	IGMT2, Indole Glucosinolate O-methyltransferase 2	Cytosol	predicted by AmiGO
AT1G21130	IGMT4, Indole Glucosinolate O-methyltransferase 4	Cytosol	predicted by AmiGO
AT5G16390	Chloroplastic acetylcoenzyme A carboxylase 1	Chloroplast	Melonek et al., 2016

a *The PubMed ID (PMID) of the publication reporting the plasma membrane (PM), extracellular or plastid proteomics data are indicated: Nguyen-Kim et al., 2016 (PMID:26572690); Mitra et al., 2009 (PMID:19334764); Heard et al., 2015 (PMID:25900983); De Michele et al., 2016 (PMID:26781341); Charmont et al., 2005 (PMID:15694452); Zhang and Peck, 2011 (PMID:21433285); Helm et al., 2014 (PMID:24361574); Melonek et al., 2016 (PMID:26987276)*.

Finally, in agreement to GLS and their degradation products being detected in the exudate metabolome (Figure 2), one of the identified apoplastic proteins (Table 1) is HOL1 (Harmless to Ozone Layer 1, AT2G43910), an enzyme involved in methylation of halides such as methyl chloride or methyl bromide that can damage ozone layer in the stratosphere. Interestingly, subsequent experiments found that HOL1 preferentially methylated thiocyanate over the halides (Rhew et al., 2003). Since thiocyanate (SCN) is the breakdown product of glucosinolate-derived isothiocyanate, SCN is likely the native HOL1 substrate. Indeed, among the three Arabidopsis HOL1-like proteins, HOL1 has the highest activity on SCN while the preferred substrate for both HOL2 and HOL3 is HS^−^ (Nagatoshi and Nakamura, 2009). Furthermore, two indole glucosinolate *O*-methyltransferases (IGMTs) were also found in the exudate proteome (Table 1), albeit these cellular enzymes have so far not been reported to be secreted. Nevertheless, the discovery of these enzymes in the apoplast is supportive of roles in modification of GLS extracellularly. In summary, our metabolome and proteome data suggest a controlled “release” of metabolites and proteins into the apoplast after sustained MPK3/MPK6 activation.

## Discussion

### MAPK-induced accumulation of metabolites and proteins in root exudates

In the current work, we used a transgenic system designed to mimic activation of two stress-inducible MAPKs, MPK3, and MPK6 (Lassowskat et al., 2014), to explore exudation of metabolites and proteins into root apoplast. Besides the metabolome and proteome changes, the responsiveness of the roots is evident from the reduction in fresh weight of the treated roots and systemic effects on leaf tissues in terms of loss in fresh weight and apparent tissue collapse (Figure 1A). Such growth inhibition phenotype is typically attributed as a trade-off between (stress-induced or in this case, MAPK-induced) defense activation and growth (Huot et al., 2014), where limited cellular resources are channeled into defense growth pathways. Supporting our postulation that MPK3/MPK6 activation may alter secretory processes, we observed differential accumulation of subsets of metabolites and proteins in the root exudates. Although there is a remote possibility that substances are being released from dying cells, this is highly unlikely as one would expect generic leakage of all cellular components rather than the observed selective accumulation (Table S2). Microscopy of the treated roots also did not reveal morphological differences between the DD and KR transgenic lines and no enhanced staining with trypan blue that would stain dead cells (Figure S2), so that all root tissues appear to be alive after 24 h of DEX treatment. Thus, the accumulation (and reduction) of detected substances in root exudates is apparently a specific and regulated MPK3/6-induced response.

### Many (putative) defense-related proteins are found among the root extracellular proteome

As no direct exposure to pathogens is involved in our experimental set-up, classical pathogenesis-related proteins are not detected. Among the detected extracellular proteins is RD21, a secreted protease reported to be involved in Arabidopsis resistance to *Botrytis cinerea* infection but not bacterial pathogens (Shindo et al., 2012). Several pathogens secrete inhibitor proteins to counteract RD21-like activities, which indicates that such proteases play important roles in plant resistance (Zhang et al., 2014; Misas-Villamil et al., 2016). Thus, the detection of RD21 supports the idea of defense regulation through MPK3/6 by increasing release of antimicrobial proteins into the extracellular milieu. Furthermore, we detected many plasma membrane proteins in the extracellular proteome (Table 1), including immune signaling scaffolds remorin and HIRs. At this stage, it is unclear if these are indeed secreted or there might be ectodomain shedding that has been seen for several animal membrane-integral receptors (Wang et al., 2002) and recently reported for the *Arabidopsis* CERK1 chitin receptor (Petutschnig et al., 2014). In either scenario, it is logical to assume that presence of such proteins (or their fragments) in the apoplast will affect extracellular immune signal transduction.

A prominent cluster of proteins in the extracellular proteome is the histone 2A group (Table 1). A simple interpretation would be that this is a sign of cell death-associated leakage (see discussion above), but again, one must question why only this sub-group and not the other nucleosome components. In fact, extracellular histones have previously been reported under conditions where no cell death is apparent (Wen et al., 2007), where histone 4 is thought to be secreted together with DNA to form part of a pea defense mechanism in the root cap slime. When this extracellular DNA is digested enzymatically, root tip resistance to infection by the pea root-rotting pathogen, *Nectria haematococca*, is compromised (Wen et al., 2009). Extracellular DNA may thus be acting as DAMPs (Krol et al., 2010) and triggering PTI in roots. Alternatively, it may be analogous to the “extracellular traps” released by animal neutrophils—a complex composed of nuclear DNA backbone associated with antimicrobial peptides, histones, and proteases, which entraps and kills pathogens (Brinkmann et al., 2004; Marin-Esteban et al., 2012). Interestingly, the amphipathic nature specific to histone 2A has been predicted to be responsible for the *in vitro* antimicrobial activities of a prawn histone 2A-like protein (Arockiaraj et al., 2013). Extracellular histones and DNA may thus be unconventional but putative defense molecules and in line with MAPK roles in activating resistance.

### Apoplastic root metabolites accumulating after MAPK activation may be allelopathic or antimicrobial compounds

In the metabolome analysis, several dipeptides were detected in the exudates. Similar dipeptides have previously been reported for *Arabidopsis* exudates (Moussaieff et al., 2013; Strehmel et al., 2014) but the biosynthetic origin of these dipeptides is currently unknown. Since they were not detected in the corresponding total roots, one may speculate that they represent breakdown products of proteins extracellularly. However, this is unlikely as otherwise dipeptides with other amino acid compositions should have been recovered. Rather, it has been shown that such dipeptides are associated with and presumably secreted by specific root epidermal cell types (Moussaieff et al., 2013). The functions of these dipeptides remain speculative but since plants possess multiple di-/tripeptide transporters that mediate their uptake, they could be sources of nitrogen nutritional acquisition for neighboring plants (Komarova et al., 2008) or could act as signaling molecules for plant-plant communication (Liu and Christians, 1994). For instance, a cyclic dipeptide found in root exudates has allelopathic properties that affect replanting of new seedlings in Chinese fir tree plantations (Chen et al., 2014). Some dipeptides are bioactive as taste modifiers (Schindler et al., 2011) or analgesics in animal systems (Perazzo et al., 2017). One of the Arabidopsis extracellular dipeptides detected in this report is YL (Figure 2). It has anxiolytic (i.e., stress reducing) activity in mice, with comparable efficacy to diazepam (better known under its trade name Valium), a drug used to treat various medical conditions like seizure/spasms, anxiety, alcohol withdrawal syndrome etc (Kanegawa et al., 2010). Such anxiolytic activity was not detected with its reverse sequence, LY, or the related YI (Note that YI was also found in the Arabidopsis exudate, Figure 2). A speculative but intriguing notion is that YL or the other dipeptides found in Arabidopsis root exudates may function to modify behavior of animal herbivores or parasites (e.g., insects or nematodes).

Another class of apoplastic compounds is the group of GLS derivatives. These were mostly degradation products of the aliphatic- compared to the indolic-type of GLS. As this transgenic system is known to strongly stimulate indolic GLS production (Lassowskat et al., 2014), one possibility is that these are rapidly metabolized once secreted into the root apoplast. GLSs and their derivatives have long been associated with plant resistance to insects or pathogens (Sotelo et al., 2015) or also in allelopathic plant-plant communications (Rivera-Vega et al., 2015). For the latter, direct treatment with GLS-containing brassica meal or extracts regulate growth of surrounding plants (Brown and Morra, 1996) and brassica plants have been used for crop rotation or intercropping as weed control in agricultural practices (Haramoto and Gallandt, 2004). With respect to pest/pathogen resistance, thiocyanates (SCNs) derived from GLS degradation are thought to be vital players. It is thus intriguing that HOL1 was found as an exudate protein after DEX treatment of the DD line (Table 1). HOL1 methylates SCN to CH_3_SCN. A direct comparison of KSCN to CH_3_SCN showed a 10- or 50-fold decrease in IC_50_ of CH_3_SCN on *Erwinia carotovora* subsp. *carotovora* or *Pseudomonas syringae* pv. *maculicola*, respectively (Nagatoshi and Nakamura, 2009). Methylated thiocyanate is thus more potent in inhibiting bacterial growth. Accordingly, the *Arabidopsis hol1* mutant showed increased susceptibility toward *P. syringae* pv. *maculicola* (Nagatoshi and Nakamura, 2009). The release of HOL1 into the root apoplastic fluid could be a MPK3/6-regulated step for converting SCN to the antimicrobially more potent CH_3_SCN.

### Concluding remarks on strength and weakness of the experimental system

In conclusion, MAPKs can regulate the composition of apoplastic root metabolites and proteins. The challenges of the current analysis include: (1) the artificial and sustained MAPK activation, which may not reflect natural kinase activity profiles when exposed to soil microbes, as well as (2) the difficulties to connect the identified apoplastic substances to a “real” biological setting. Additionally, a criticism of root exudate analyses relying on soil-less hydroponics (as used here) or aeroponics systems is that it may not reflect exudate profiles in the natural environment. Furthermore, volatile organic compounds may be overlooked. However, effects of microorganisms colonizing the rhizosphere or environmental factors (soil properties, temperature, oxygen status etc.) complicate the analysis of root exudates. For instance, comparison to a previous analysis of root exudates in plants colonized by the mutualistic *Piriformaspora indica* fungus (Strehmel et al., 2016) showed only a partial overlap of the exudated metabolites/dipeptides. While some metabolites (e.g., 7-MeSO-heptyl-CN, 8-MeSO-octyl-CN, 8-MeSO-octyl-CONH_2_) showed similar increase in exudates in both systems, others (e.g., 3-Carboxy-1,2,3,4-tetrahydro-beta-carboline, thymidine) were reduced during *P. indica* cocultivation but increased in the current experimental setup. A cell wall elicitor fraction isolated from *P. indica* can activate MAPKs in Arabidopsis roots (Vadassery et al., 2009). Thus, exposure to *P. indica* is expected to lead to MAPK signaling. The discrepancy of the exudated metabolite profile illustrates the complication of “real” plant-microbe studies. Symbionts or pathogenic microbes may metabolize the compounds or induce/repress root exudation (reviewed in Van Dam and Bouwmeester, 2016). An advantage of the transgenic system used in the current study is the reduction of confounding effects of multiple microbe-induced signaling pathways; specifically it represents a “stripped down” version to focus on exudation processes controlled by MAPKs. We show that MAPK activities indeed regulate appearance of metabolites or proteins in root exudates. Whether this truly represents increased exudation must still be proven. To address this and unravel defense metabolite exudation processes, future experimental design will include mimicking MAPK activation profile occurring during natural interaction with soil microbes (in terms of temporal dynamics or induction strength through the DEX-inducible system), which can further be combined with studies involving secretory pathway inhibitors or incorporating genetic mutants of specific biosynthesis, regulatory or secretory pathways-of-interest.

## Data availability

The Metabolomics data is available in the Metabolights repository under the accession number (MTBLS441, http://www.ebi.ac.uk/metabolights/MTBLS441). The mass spectrometry proteomics data have been deposited to the ProteomeXchange Consortium via the PRIDE partner repository with the dataset identifier PXD006328 and DOI 10.6019/PXD006328.

## Author contributions

NS, DS, and JL designed the project. NS, WH, PM, and SK performed the experiments. NS, PM, and WH acquired the metabolomics and proteomics data, respectively. NS, WH, SM, and JL analyzed the data. NS, SM, DS, and JL wrote the manuscript. All authors have read the final manuscript.

### Conflict of interest statement

The authors declare that the research was conducted in the absence of any commercial or financial relationships that could be construed as a potential conflict of interest. The reviewer SCP and handling Editor declared their shared affiliation, and the handling Editor states that the process met the standards of a fair and objective review.
