# Supplementary material for: Stress-Related Mitogen-Activated Protein Kinases Stimulate the Accumulation of Small Molecules and Proteins in Arabidopsis thaliana Root Exudates
Source: Front Plant Sci. 2017 Jul 21;8:1292. doi: 10.3389/fpls.2017.01292 (PMC5520323; doi:10.3389/fpls.2017.01292)
Supplement: Supplementary file 1 [file DataSheet1.pdf]

Supplementary Material

Stress-Related Mitogen-Activated Protein Kinases Stimulate the Accumulation of Small Molecules and Proteins in Arabidopsis thaliana Root Exudates

Nadine Strehmel, Wolfgang Hoehenwarter, Susann Mönchgesang, Petra Majovsky, Sylvia Krüger, Dierk Scheel, Justin Lee

*** Correspondence:** Corresponding Author: dscheel@ipb-halle.de and jlee@ipb-halle.de

1 Supplementary Methods

Additional details of the LC/MS- based metabolite profiling

UPLC settings:

- Solvents: A (0.1% formic acid in water); B (0.1% formic acid in acetonitrile)
- Column: HSS T3 column (100 × 1.0 mm, particle size 1.8 µm, Waters)
- Gradient: 0–1 min, isocratic 95% A, 5% B; 1–10 min, linear from 5 to 60% B; 10–12 min, isocratic 95% B; 12–14 min, isocratic 5% B
- Flow rate: 150 µL min^{-1}
- Temperature: 40°C

Mass spectrometer:

- *m/z* 100–1000 in positive and negative ion mode
- nebulizer gas: nitrogen, 1.6 bar
- dry gas: nitrogen, 6 l/min, 190°C
- capillary, −5000 V (+4000 V); end plate offset, −500 V; funnel 1 RF, 200 Vpp; funnel 2 RF, 200 Vpp;
- hexapole RF: 100 Vpp
- quadrupole ion energy: 3 eV
- collision gas: argon; collision energy, 3 eV for ESI+ and 10 eV for ESI-; collision RF 200/400 Vpp (timing 50/50); transfer time, 70 µs; pre pulse storage, 5 µs; pulser frequency, 10 kHz

2. Figure S1

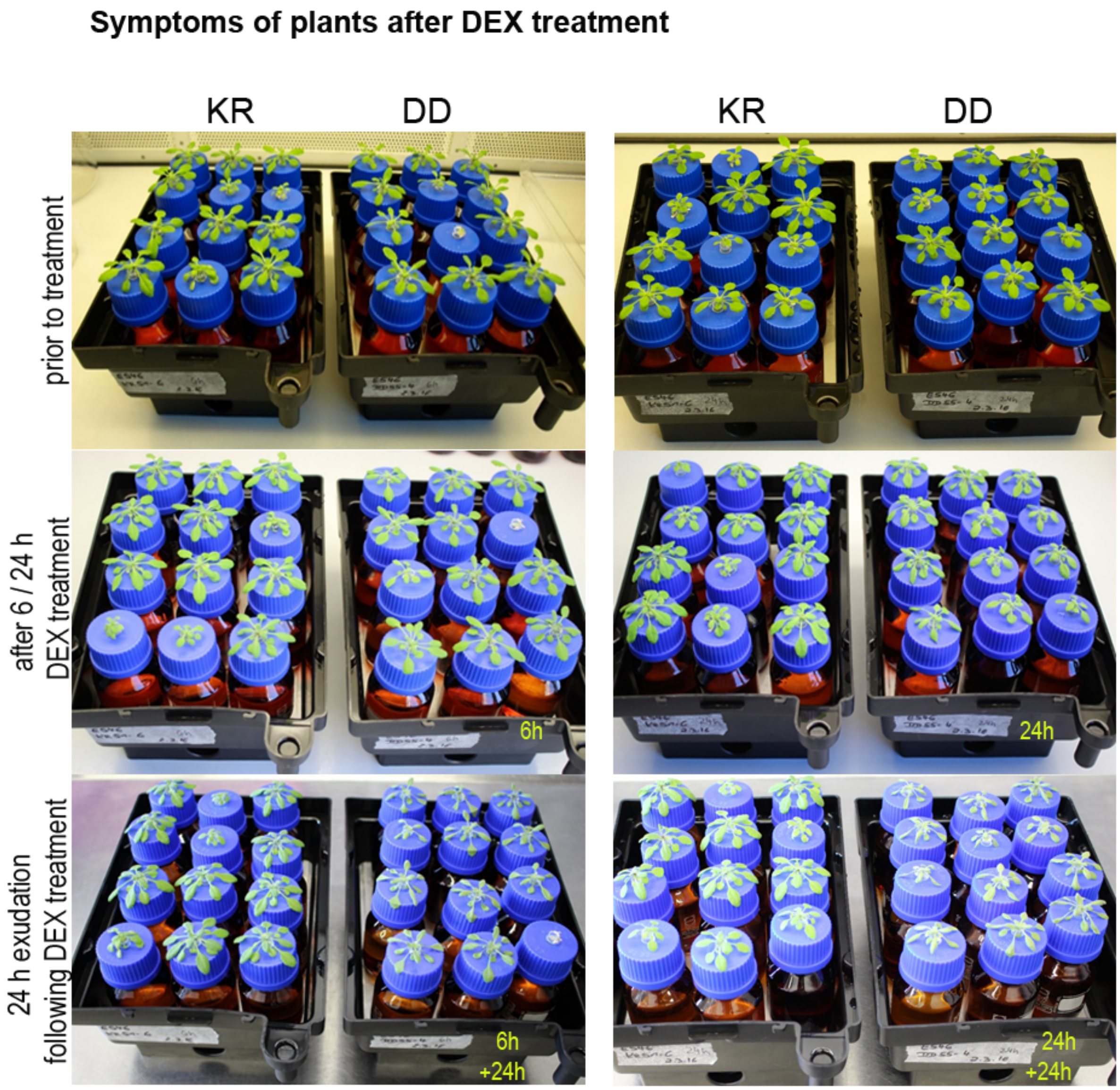
Symptoms of plants after DEX treatment
KR
DD
KR
DD
prior to treatment
after 6 / 24 h DEX treatment
24 h exudation following DEX treatment
6h
24h
6h +24h
24h +24h

Figure S2

Trypan-blue staining of roots (after treatment with DEX for 24 h).

Note that only the central vasculature is stained. All other cells/tissues appear morphologically intact and are therefore presumably alive.

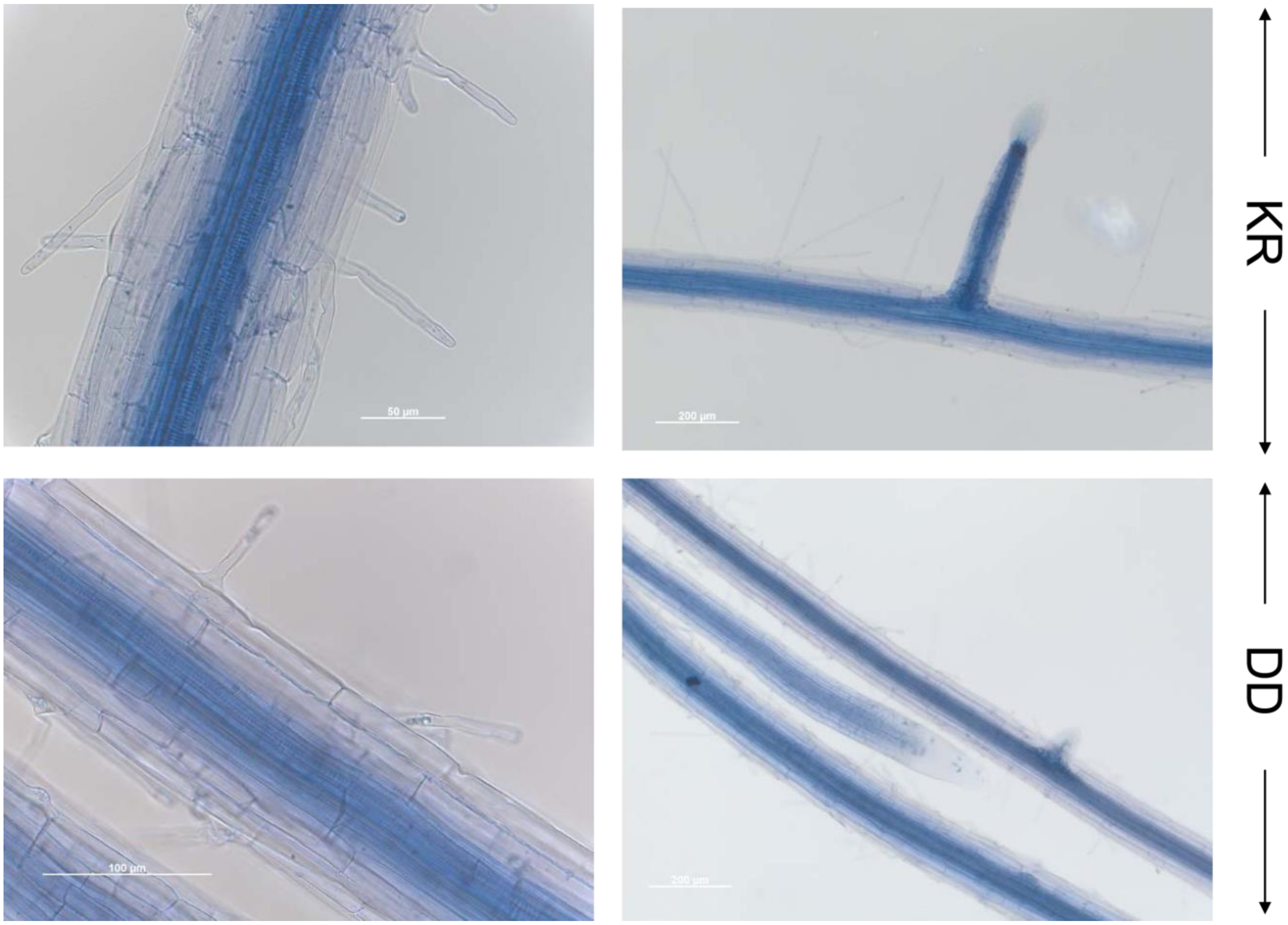

3. Supplementary Tables (in Excel format)

Supplementary Table S1: Analytical Data of Roots after 6h or 24h dexamethasone treatment (and subsequent exudation for 24h into LC/MS-grade water).

Supplementary Table S2: Analytical Data of Root Exudates collected after 6h as well as 24h dexamethasone treatment and subsequent exudation for 24h into LC/MS-grade water.

Supplementary Table S3: Analytical characterization (MS/MS) of metabolites.

Supplementary Table S4: List of up-regulated (red) and down-regulated (green) proteins detected in the exudates of dexamethasone-treated DD line compared to the KR line.
